# Short-term effect of humidified high nasal flow oxygen in critically ill patients

**DOI:** 10.1186/cc10744

**Published:** 2012-03-20

**Authors:** F Van Beers, A Van Hees, J Van Rosmalen, D Ramnarain

**Affiliations:** 1 St Elisabeth Hospital Tilburg, the Netherlands

## Introduction

Recently, humidified high-flow nasal cannula oxygen (HFNC) has gained popularity in treating patients with acute respiratory insufficiency. Studies have shown that HFNC generates a low level of positive airway pressure, reduction of airway resistance and flushes nasopharyngeal dead space leading to less work of breathing. However, in which type of patient HFNC could be of benefit, the short-term as well as long-term effects, tolerance and outcome are unknown. We used HFNC in a variety of patients. We evaluated the short-term effect of HFNC.

## Methods

We retrospectively studied respiratory, oxygen-derived and hemodynamic parameters before and 1 hour after start of HFNC in 50 patients during the past 12 months. All patients were treated in a mixed medical, surgical, neurological ICU of a teaching hospital. The HFNC used consisted of an air-oxygen blender with adjustable FiO_2 _(0.21 to 1.0), delivering a modifiable gas flow up to 60 l/minute (Optiflow; Fisher & Paykel, Auckland, New Zealand).

## Results

Fifty patients were included, 29 men and 21 women, mean age 65 ± 14, mean APACHE II score on admission 19 ± 5.9. The mean duration of HFNC was 22 ± 21 hours. Indications for HFNC could be divided into five categories: (1) no acceptance of noninvasive positive pressure ventilation (NPPV) (*n *= 8), (2) weaning from NPPV, (3) hypoxia (*n *= 14), (4) respiratory distress/discomfort (*n *= 9), and (5) other (*n *= 5). Despite the use of HFNC, in 15 patients intubation was unavoidable; group 1, *n *= 8, group 3, *n *= 6, group 4, *n *= 1. Oxygen saturation increased from 91 ± 7.2 to 97.5 ± 1.7 (*P *≤0.05). PaO_2_/FiO_2 _ratio increased from 140 ± 79.1 to 169.8 ± 68 (*P *≤0.05). PCO_2 _decreased from 6.5 ± 3.0 to 6.2 ± 2.9 mmHg (*P *≤0.05). No significant differences were seen in heart rate, blood pressure and respiratory rate. Ten patients died, in eight patients of which the policy was not to reanimate and not to be intubated due to extensive comorbidity. Two patients died during treatment in the ICU due to underlying disease.

## Conclusion

We used HFNC therapy for a variety of indications. In 70% of our study population HFNC was successful. Oxygen-derived parameters significantly increased after 1 hour of HFNC. HFNC was successful and well tolerated in patients weaning from NPPV. After noncompliance of NPPV in 42% of patients in our population, intubation could be avoided with the use of HFNC.

